# Nervonic acid improves fat transplantation by promoting adipogenesis and angiogenesis

**DOI:** 10.3892/ijmm.2024.5432

**Published:** 2024-09-30

**Authors:** Jae Hoon Song, Sun Jeong Kim, Soojin Kwon, Su Yeon Jeon, Sang Eon Park, Suk-Joo Choi, Soo-Young Oh, Hong Bae Jeon, Jong Wook Chang

**Affiliations:** 1 Cell and Gene Therapy Institute, ENCell Co., Ltd., Seoul 06072, Republic of Korea; 2 Cell and Gene Therapy Institute, Samsung Medical Center, Seoul 06351, Republic of Korea; 3 Department of Obstetrics and Gynecology, Samsung Medical Center, Seoul 06351, Republic of Korea; 4 Department of Health Sciences and Technology, Samsung Advanced Institute for Health Sciences and Technology (SAIHST), Sungkyunkwan University, Seoul 06355, Republic of Korea

**Keywords:** nervonic acid, fat transplantation, adipogenesis, angiogenesis, inflammation, adjuvant, adipocyte viability

## Abstract

Adipose tissue engraftment has become a promising strategy in the field of regenerative surgery; however, there are notable challenges associated with it, such as resorption of 50-90% of the transplanted fat or cyst formation due to fat necrosis after fat transplantation. Therefore, identifying novel materials or methods to improve the engraftment efficiency is crucial. The present study investigated the effects of nervonic acid (NA), a monounsaturated very long-chain fatty acid, on adipogenesis and fat transplantation, as well as its underlying mechanisms. To assess this, NA was used to treat cells during adipogenesis *in vitro*, and the expression levels of markers, including PPARγ and CEBPα, and signaling molecules were detected through reverse transcription-quantitative PCR and western blotting. In addition, NA was mixed with fat grafts in *in vivo* fat transplantation, followed by analysis through Oil Red O staining, hematoxylin & eosin staining and immunohistochemistry. It was demonstrated that NA treatment accelerated adipogenesis through activation of the Akt/mTOR pathway and inhibition of Wnt signaling. NA treatment enriched the expression of Akt/mTOR signaling-related genes, and increased the expression of genes involved in angiogenesis and fat differentiation in human mesenchymal stem cells (MSCs). Additionally, NA effectively improved the outcome of adipose tissue engraftment in mice. Treatment of grafts with NA at transplantation reduced the resorption of transplanted fat and increased the proportion of perilipin-1^+^ adipocytes with a lower portion of vacuoles in mice. Moreover, the NA-treated group exhibited a reduced pro-inflammatory response and had more CD31^+^ vessel structures, which were relatively evenly distributed among viable adipocytes, facilitating successful engraftment. In conclusion, the present study demonstrated that NA may not only stimulate adipogenesis by regulating signaling pathways in human MSCs, but could improve the outcome of fat transplantation by reducing inflammation and stimulating angiogenesis. It was thus hypothesized that NA could serve as an adjuvant strategy to enhance fat engraftment in regenerative surgery.

## Introduction

Adipose tissue transplantation, pioneered in 1889, is considered an important regenerative strategy for soft tissue augmentation of the breast or face in patients with soft tissue deficiency, as well as for cosmetic surgery purposes (1). For transplantation, autologous or allogeneic adipose tissue is harvested from selected donor sites, isolated into viable adipocytes and adipose-derived mesenchymal stem cells (AD-MSCs) by processing, and then grafted to the target site (1,2). Owing to the availability of abundant donor tissues, easy procedures and relatively low immunogenicity, adipose tissue has become a promising filling material for reconstruction (3). For successful engraftment of the transplanted tissue in recipients, it is crucial to create and maintain an integral environment within the graft through various strategies, including neovascularization and *de novo* adipogenic differentiation (4,5). However, tissue damage during the transplantation stage and failures in reorganizing these environments within adipose tissue after transplantation can trigger the collapse of the graft construct, resulting in major side effects, such as a 50-90% resorption of the total injection volume, necrosis and cyst formation (2,5,6). Although various approaches have been utilized for stabilizing the overall integrity of adipose grafts, including biofabrication of materials mimicking the characteristic properties of fat tissue and the stimulation of stem cells to differentiate into viable adipocytes, these methods have shown limited effectiveness when applied alone (5,7-9). Therefore, it has become increasingly necessary to discover new adjuvant materials or methods that can improve the efficiency of adipose tissue engraftment and provide a viable environment after grafting.

Nervonic acid (NA) is a monounsaturated very long-chain fatty acid primarily localized in mammalian nerve tissues in the brain (10). Because of its role in maintaining and forming the main component of myelinated nerves, NA has been studied as a biomarker or therapeutic candidate in various neuronal diseases, such as Alzheimer's disease and depression (10,11). Growing evidence over the last few decades has indicated that NA is also related to fatty acid metabolism, affecting metabolic phenotypes, and may thus be considered a novel diagnostic marker of metabolic disorders. Keppley *et al* (12) reported that the treatment of obese mice with NA could improve energy metabolism and promote sphingolipid recovery, indicating that NA may influence adipocytic characteristics. Recently, our previous study discovered that NA treatment promoted adipogenesis and elevated several lipid metabolism-associated genes in human MSCs (13). Based on these results, it may be hypothesized that NA treatment could have adjuvant functions in adipogenic differentiation and fat engraftment.

The present study investigated the adjuvant effects of NA on adipogenesis and adipose tissue engraftment, as well as its underlying mechanisms, using both *in vitro* and *in vivo* experiments. To address this, MSCs were treated with NA during adipogenic differentiation, after which, the affected signaling pathways were identified. Additionally, adipose tissue grafts were transplanted alongside NA and the factors affecting adipocyte viability were investigated. The findings indicated that NA may be a potential agent for improving the outcomes of adipose tissue engraftment.

## Materials and methods

### Isolation and culture of MSCs

Human Wharton's jelly, placenta, umbilical cord blood and adipose tissue samples were obtained from women after they had given birth at Samsung Medical Center (Seoul, South Korea) between September 2016 and September 2024, and were used to isolate MSCs. Each type of tissue was obtained from one individual. All participants provided written informed consent, and the procedures were approved by the Institutional Review Board of Samsung Medical Center (approval no. 2016-07-102-001; Seoul, South Korea). AD-MSCs were isolated from adipose tissues according to a previously reported method (14). Placenta-derived MSCs were isolated from the placenta, following a previously described method (15). Umbilical cord blood-derived MSCs were isolated from umbilical cord blood, as previously reported (16). Wharton's jelly-derived MSCs (WJ-MSCs) were isolated from the separated umbilical cord, as previously described (17). Human bone marrow-derived MSCs (cat. no. PT-2501) were purchased from Lonza Group, Ltd. All MSCs were cultured in α-modified minimum essential medium supplemented with 10% fetal bovine serum and 50 *μ*g/ml gentamicin (all from Gibco; Thermo Fisher Scientific, Inc.) at 37°C in a 5% CO_2_ incubator. MSCs at passage 7 were used in the experiments.

### Cytotoxicity measurement

Cytotoxicity was measured using the Cell Counting Kit-8 (CCK-8; Dojindo Molecular Technologies, Inc.), according to the manufacturer's instructions. AD-MSCs were seeded at a density of 1×10^3^ cells/well in 96-well culture plates and were incubated overnight. AD-MSCs were treated with different concentrations of NA (0, 40, 80, 120, 160, 200, 240 and 280 *μ*M) or an equal volume of dimethyl sulfoxide (DMSO) as a control, and then incubated for 3 or 7 days at 37°C. For treatment, NA [≥99% (capillary GC); cat. no. 506-37-6; MilliporeSigma] was prepared as a 50 mM stock solution in DMSO (MilliporeSigma). Subsequently, they were incubated with 10 *μ*l CCK-8 at 37°C for 2 h. An equal volume of medium without cells was used as a blank. The absorbance was measured at 450 nm using a microplate reader (Thermo Scientific™ Multiskan SkyHigh Microplate Spectrophotometer; cat. no. A51119600C; Thermo Fisher Scientific, Inc.).

### Adipogenic differentiation assay

MSCs were seeded in 6-well culture plates at a density of 1×10^5^ cells/well and were incubated until the cells reached 80-90% confluence. For adipogenic differentiation, MSCs were cultured in an adipogenesis differentiation medium (StemPro™ Adipogenesis Differentiation kit; cat. no. A1007001; Gibco; Thermo Fisher Scientific, Inc.) containing different concentrations of NA (0, 40, 80, 120, 160, 200, 240 and 280 *μ*M), according to the manufacturer's instructions. The control group was treated with an equal volume of DMSO in the same differentiation medium. After 7, 14 and 21 days, the differentiated cells were fixed with 4% paraformaldehyde (Biosaesang) for 10 min and stained with Oil Red O solution (MilliporeSigma) for 30 min at 25°C. Subsequently, lipid droplets were observed under a light microscope (CKX41; Olympus Corporation). Oil Red O was quantified by extraction in 100% isopropyl alcohol and the optical density was measured at 540 nm.

### RNA isolation and reverse transcription-quantitative PCR (RT-qPCR)

Total RNA was obtained from the differentiated AD-MSCs using TRIzol^®^ reagent (Invitrogen; Thermo Fisher Scientific, Inc.). Next, complementary DNA (cDNA) was synthesized from the total RNA using SuperScript IV Reverse Transcriptase (Invitrogen; Thermo Fisher Scientific, Inc.), according to the manufacturer's instructions, and was further purified using a QIAquick PCR Purification kit (Qiagen GmbH). qPCR was performed by adding cDNA to a mixture containing the respective primers and SYBR green master mix (Applied Biosystems; Thermo Fisher Scientific, Inc.) using the QuantiStudio™ 6 Flex Real-time PCR system (Applied Biosystems; Thermo Fisher Scientific, Inc.). The qPCR conditions were as follows: Pre-denaturation at 50°C for 2 min and 95°C for 10 min; followed by 40 cycles of amplification at 95°C for 30 sec, 60°C for 30 sec and 72°C for 30 sec; and a melting curve stage at 95°C for 15 sec, 60°C for 1 min and 95°C for 15 sec. The specific primer sequences are listed in Table I. *GAPDH* was used as the housekeeping gene, and the results were analyzed using the 2^−ΔΔCq^ method (18).

### Antibodies and reagents

The following primary antibodies were used for western blotting and immunohistochemistry experiments: PPARγ (cat. no. 2435), CEBPα (cat. no. 2295), adiponectin (cat. no. 2789), phosphorylated (p)-Akt (Ser473; cat. no. 9271), Akt (cat. no. 9272), p-mTOR (Ser2448; cat. no. 5536), mTOR (cat. no. 2983), GSK3β (cat. no. 9315), p-β-catenin (Ser33/37/Thr41; cat. no. 9561), β-catenin (cat. no. 9562), p-Smad1/5 (Ser463/465; cat. no. 9516), Smad1 (cat. no. 9743), p-ERK1/2 (Thr202/Tyr204; cat. no. 9101), ERK1/2 (cat. no. 9102), perilipin-1 (cat. no. 9349) and CD31 (cat. no. 77699), which were purchased from Cell Signaling Technology, Inc.; and lipoprotein lipase (LPL; cat. no. sc-373759) and β-actin (cat. no. sc-47778), which were obtained from Santa Cruz Biotechnology, Inc. HRP-conjugated anti-rabbit (cat. no. abc-5003) or anti-mouse (cat. no. abc-5001) secondary antibodies (AbClon) were used as secondary antibodies in the western blot analysis.

### Western blot analysis

To determine the levels of adipogenic markers in AD-MSCs, the cells were cultured for 14 days in adipogenesis differentiation medium with either DMSO or NA. The cells were then lysed with RIPA buffer (Biosaesang) + protein inhibitor cocktail (GenDEPOT) + EDTA (GenDEPOT) and centrifuged at 18,000 × g for 30 min at 4°C to collect the lysate. Protein concentration was quantified using the Bradford assay (Bio-Rad Laboratories, Inc.). Protein extracts (10 *μ*g/lane) were then separated by SDS-PAGE on 4-15% polyacrylamide gels (Bio-Rad Laboratories, Inc.). Subsequently, the separated proteins were transferred onto nitrocellulose or polyvinylidene fluoride membranes, which were blocked with 5% skim milk (Difco; BD Biosciences) or 5% bovine serum albumin (BSA) (Thermo Fisher Scientific, Inc.) in TBS-0.2% Tween-20 (TBS-T) and were gently shaken for 60 min at 25°C. The membranes were then incubated with primary antibodies (1:1,000 dilutions in 5% skim milk or 5% BSA) overnight at 4°C with gentle shaking. After three washes in TBS-T (10 min/wash) at 25°C, the membranes were incubated with HRP-conjugated anti-rabbit or anti-mouse secondary antibodies (1:5,000 dilutions in 5% skim milk or 5% BSA) at 25°C for 1 h with gentle shaking. Finally, the membranes were washed three times with TBS-T (10 min/wash), were treated with ECL solution (Bio-Rad Laboratories, Inc.) for 1 min, were visualized using an Amersham™ Imager 600 (Cytiva), and were semi-quantified using ImageJ 1.53 software (National Institutes of Health). The primary and secondary antibodies used in western blotting are aforementioned.

### mRNA sequencing

Total RNA was isolated for mRNA sequencing from differentiating AD-MSCs at 7, 14 and 21 days using TRIzol^®^ reagent (cat. no. 15596018; Invitrogen; Thermo Fisher Scientific, Inc.) following the manufacturer's instructions. mRNA sequencing was conducted by Ebiogen Inc. RNA quality was assessed using the TapeStation4000 System (Agilent Technologies, Inc.) and RNA was quantified using an ND-2000 spectrophotometer (Thermo Fisher Scientific, Inc.). NEBNext Ultra II Directional RNA-Seq kit (cat. no. E7760L; New England BioLabs, Inc.) was used to prepare the sequencing libraries from total RNA, and each step was performed using reagents included within the kit. The mRNA was further isolated from total RNA using a Poly(A) RNA Selection kit (cat. no. LEX-157; Lexogen GmbH). Subsequently, cDNA was synthesized from the isolated mRNAs (10 min at 25°C; 15 min at 42°C; 15 min at 70°C for first strand cDNA and 1 h at 16°C for second strand cDNA), and removal of RNA was followed by random-primed synthesis of the complementary strand. The cDNA was sheared according to the manufacturer's instructions of the NEBNext Ultra II Directional RNA-Seq kit. Indexing was performed using Illumina indices 1-12. Library enrichment was performed using PCR. The libraries were checked for quality control using TapeStation HS D1000 Screen Tape (Agilent Technologies, Inc.) to evaluate the mean fragment size. Quantification was performed on the StepOne Real-Time PCR System (Thermo Fisher Scientific, Inc.) using a random primer and NEBNext Ultra II Q5 Master Mix (included in the NEBNext Ultra II Directional RNA-Seq kit). High-throughput sequencing (paired-end 100 bp sequencing) was performed with a loading concentration of 320 pM using NovaSeq 6000 (Illumina, Inc.).

### Data analysis

First, FastQC was used for quality control of the raw sequencing data (19). Adapter and low-quality reads (<Q20) were removed using FASTX_Trimmer (20) and BBMap (21). Trimmed reads were mapped to the reference genome using TopHat (22). The read counts were calculated using Cufflinks (23) and the fragments per kb per million reads (FPKM) values were estimated using Cuffdiff. The FPKM values were normalized using Cuffdiff geometric normalization. Data mining and graphic visualization were performed using ExDEGA ver 4.0.3 (ebiogen, Inc.). Data for each sample are available at the Gene Expression Online (GEO) under the accession number GSE269065 (https://www.ncbi.nlm.nih.gov/geo/query/acc.cgi?acc=GSE269065).

The differentially expressed genes (DEGs) between the DMSO and NA groups that showed a fold change >1.5 and P<0.05 were selected and further analyzed using ExDEGA software and DAVID Bioinformatics Resource 6.8 (NCBI online bioinformatics program) (https://david.ncifcrf.gov/tools.jsp) (24,25) to identify the functions of the selected genes and Gene Ontology. Adjusted P≤0.05 (Fisher's Exact test) was used to select related functional annotations. The principal component analysis (PCA) and the heatmaps of gene expression were generated using Excel 2016 (Microsoft) and ExDEGA software. Additionally, enriched signaling pathways related to the selected DEGs were analyzed using the online Kyoto Encyclopedia of Genes and Genomes (KEGG) mapper tool (https://www.kegg.jp/kegg/mapper.html) (26,27).

### In vivo fat transplantation

Fat transplantation experiments were performed using 5-month-old C57BL/6 mice (ORIENT BIO, Inc.) as both adipose tissue donors and recipients. Both male and female mice were used randomly, which weighed 22-28 g, and had free access to food and water. A total of 33 mice were used for the experiments. Specifically, 15 mice (10 donors, 5 recipients) were used for fat graft measurements and gene expression analysis, and 18 mice (12 donors, 6 recipients) were used for histological analysis. Mice for the experiments were housed in individually ventilated cages with a maximum of five mice per cage. The housing room was air-conditioned with 100% HEPA-filtration. The internal temperature and humidity were maintained at 20-26°C and 30-70%, respectively, and mice were kept under a 12-h light/dark cycle. For fat extraction, mice were anesthetized with 2% isoflurane and allogeneic adipose tissues for transplantation were extracted from the peritoneal fat of mice after laparotomy. The extracted adipose tissues were finely chopped and centrifuged at 2,095 × g for 15 min at 25°C. Subsequently, the upper and lower fractions containing lipids and blood, respectively, were removed to collect pure adipose tissue for engraftment. Thereafter, adipose tissue was transplanted underneath the skin of the back on both sides: Adipose tissue (300 *μ*l) mixed with 160 *μ*M NA was transplanted into the right side of the back, whereas adipose tissue (300 *μ*l) mixed with the same volume of DMSO was transplanted into the left side. For transplantation, mice were anaesthetized with 2% isoflurane. After transplantation, the health and well-being of the mice were assessed three times a week and transplants were harvested and analyzed after 5 weeks. At transplant harvest, the mice were euthanized using CO_2_ (35% volume/min) in a chamber for 3-4 min. The death of the mice was confirmed through the cessation of heartbeat and loss of reflex. The graft volume was calculated using the formula reported by Tomayko and Reynolds (28). For histological analysis, the obtained grafts were frozen and sectioned (12 *μ*m) for Oil Red O staining using NovaUltra Oil Red O Stain kit (cat. no. IW-3008; IHC World LLC) according to the manufacturer's instructions. In addition, grafts were embedded in paraffin and sectioned (4 *μ*m) for immunohistochemistry and hematoxylin & eosin (H&E) staining.

The present study was reviewed and approved by the Institutional Animal Care and Use Committee of the Research Institute for Future Medicine (RIFM), belonging to Samsung Medical Center (approval nos. 20230316003 and 20240522001). The RIFM is an Association for Assessment and Accreditation of Laboratory Animal Care International-accredited facility that abides by the guidelines of the Institute of Laboratory Animal Research (29).

### Immunohistochemistry

For immunohistochemistry, adipose tissues from mice were embedded in paraffin and sectioned (4 *μ*m) after fixation in 4% paraformaldehyde for 36 h at 25°C. First, the samples were deparaffinized in a gradually decreasing concentration of ethanol and were permeabilized using xylene. Antigen retrieval was then performed using Target Retrieval Solution, Citrate pH 6 (Dako; Agilent Technologies, Inc.), washed and blocked in Dako REAL Peroxidase-Blocking Solution (Dako; Agilent Technologies, Inc.). After washing with PBS three times (5 min/wash), the samples were treated with primary antibodies (1:200) at 4°C. The next day, the primary antibodies were removed, and the samples were washed three times with PBS. They were then treated with Dako Envision + System-HRP Labelled Polymer Anti-Rabbit Antibody (cat. no. K4003, Dako; Agilent Technologies, Inc.) for 1 h and stained with Dako Liquid DAB (Dako; Agilent Technologies, Inc.) for 1 min at 25°C. Finally, the samples were stained with hematoxylin for nuclear staining for 4 sec at 25°C, then dehydrated and cleared before observation. Aperio AT2 (Leica Biosystems) was used for observation, and staining was semi-quantified using ImageJ 1.53 software or QuPath 0.4.4. software (30). The aforementioned primary antibodies were used for immunohistochemistry.

### H&E staining

For H&E staining, adipose tissues from mice were embedded in paraffin and sectioned (4 *μ*m) after fixation in 4% paraformaldehyde for 36 h at 25°C. First, 4-*μ*m paraffin-embedded samples were permeabilized using Histoclear (National Diagnostics) for 9 min (3 min × 3), and were rehydrated using 100 and 95% ethanol. The samples were then stained with hematoxylin for 8 min, exposed to HCl for 1 sec and eosin for 40 sec at 25°C, and washed with PBS between each step. Subsequently, the sections were dehydrated with 95 and 100% ethanol, and were treated with Histoclear. Finally, the samples were mounted, and staining was observed using Aperio AT2.

### Graphical illustrations

Graphical illustrations presented in the present study were generated using BioRender (https://www.biorender.com/).

### Statistical analysis

All data are presented as the mean ± SD (n≥3). Excel 2016 (Microsoft Corporation) and GraphPad Prism for Windows ver. 10 (Dotmatics) were used for all analyses. Statistical comparisons between two groups were performed using unpaired Student's t-test, and those between three or more groups were performed using one-way ANOVA followed by Tukey's post hoc test. P<0.05 was considered to indicate a statistically significant difference.

## Results

### NA promotes adipogenesis of human AD-MSCs

**Figure 1:**
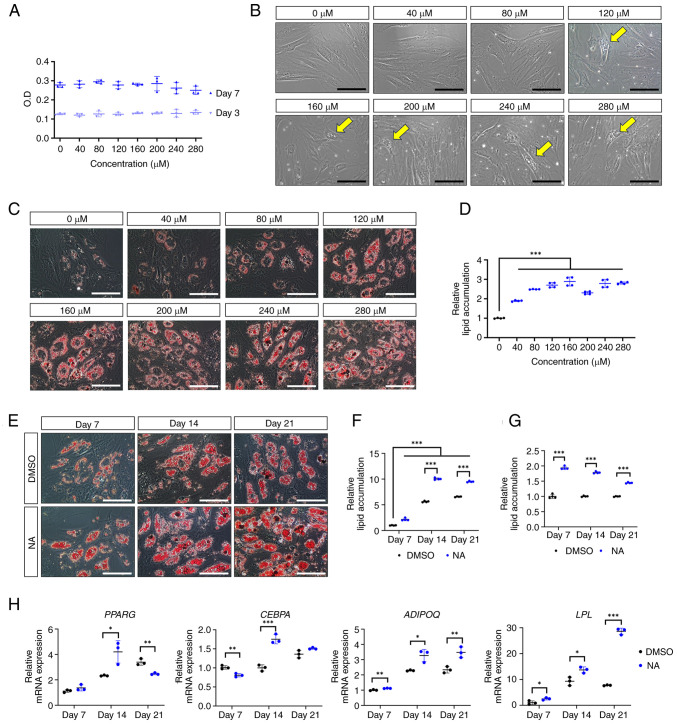
Effect of NA on adipogenesis. (A) Cell viability of AD-MSCs as measured by O.D. values at day 3 and 7 after treatment with DMSO or serial doses of NA. (B) Representative images of AD-MSCs after 72 h of NA treatment. Yellow arrows indicate the oil droplets formed after NA treatment in the normal proliferation condition. Scale bars: 100 *μ*m. (C) Representative images of Oil Red O staining of AD-MSCs on day 7 after adipogenic differentiation and treatment with DMSO or NA. Scale bars: 100 *μ*m. (D) Relative amounts of lipid accumulation after 7 days of adipogenic differentiation in cells treated with DMSO or NA. Statistical significance was assessed by one-way ANOVA: ^***^P<0.001. (E) Representative images of Oil Red O staining of AD-MSCs after 7, 14 and 21 days of adipogenic differentiation and treatment with DMSO or 160 *μ*M NA. Scale bars: 100 *μ*m. (F and G) Relative amounts of lipid accumulation after 7, 14 and 21 days of adipogenic differentiation and treatment with DMSO or 160 *μ*M NA. (F) Increase in lipid accumulation by differentiation period and NA treatment. Relative lipid accumulation was normalized to the DMSO group at day 7. Statistical significance was assessed by one-way ANOVA: ^***^P<0.001. (G) Change in lipid accumulation at each differentiation timepoint (7, 14 and 21 days) in the NA group versus the DMSO group. Relative lipid accumulation was normalized to the DMSO group at each time point. Statistical significance was assessed by unpaired Student's t-test: ^***^P<0.001. (H) Reverse transcription-quantitative PCR analysis of adipogenic markers after 7, 14 and 21 days of adipogenic differentiation and treatment with DMSO or 160 *μ*M NA. Statistical significance was assessed by unpaired Student's t-test: ^*^P<0.05, ^**^P<0.01 and ^***^P<0.001. Data are presented as the mean ± SD. AD-MSCs, adipose-derived mesenchymal stem cells; DMSO, dimethyl sulfoxide; *LPL*, lipoprotein lipase; NA, nervonic acid; O.D., optical density.

To determine the optimal concentration of NA that induces adipogenesis in AD-MSCs, the formation of oil droplets was examined in AD-MSCs following treatment with various concentrations of NA. First, the cytotoxicity of NA was determined in AD-MSCs using a CCK-8 assay. The optical density of AD-MSCs was measured on days 3 and 7 post-treatment with DMSO or several concentrations of NA (40-280 *μ*M). The viability of AD-MSCs was not significantly altered at day 3 or 7 after NA treatment compared with that in the control group (Fig. 1A). Therefore, NA was confirmed to be non-toxic to AD-MSCs. Notably, AD-MSCs treated with ≥120 *μ*M NA exhibited partial formation of oil droplets at day 3 in culture conditions, indicating that NA is a potential adipogenic inducer (Fig. 1B). Based on these results, AD-MSCs were treated with DMSO or NA during differentiation into the adipogenic lineage for 7 days, and the degree of lipid accumulation was determined using Oil Red O staining. Concentrations of NA up to 160 *μ*M enhanced lipid accumulation in AD-MSCs in a dose-dependent manner (Fig. 1C and D). Thus, 160 *μ*M was considered the optimal concentration of NA for promoting adipogenesis in AD-MSCs and was used in subsequent experiments.

Next, the effect of NA on adipogenic differentiation according to different exposure periods was assessed. Adipogenic conditions were induced for 7, 14 and 21 days, and lipid accumulation was measured using Oil Red O staining (Fig. 1E). AD-MSCs treated with NA exhibited sufficient differentiation from day 14 onward, resulting in a significant increase in lipid accumulation compared with the DMSO control group (Fig. 1F). However, lipid accumulation caused by NA showed the greatest difference on day 7 relative to the control (day 7, 1.94±0.05 fold change; day 14, 1.78±0.03 fold change; day 21, 1.45±0.02 fold change) (Fig. 1G). Because NA promoted the adipogenic differentiation of AD-MSCs, the present study further investigated whether NA could influence the induction of adipogenic genes. RT-qPCR results showed that the master transcription factors *PPARG* and *CEBPA* exhibited the highest upregulation on day 14 after NA treatment, whereas the mature adipocyte markers *LPL* and *ADIPOQ* were upregulated the most on day 21 after NA treatment (Fig. 1H). These results indicated that NA treatment was effective in accelerating the adipogenic process in AD-MSCs.

The effect of NA on MSCs from different sources was further verified on days 7, 14 and 21. Treatment with NA significantly enhanced lipid accumulation in MSCs isolated from the umbilical cord blood, placenta, Wharton's jelly and bone marrow (Fig. S1), demonstrating that NA had a universal effect on adipogenesis in MSCs, irrespective of their origin.

### NA regulates the adipogenic process via the Akt/mTOR and Wnt pathways

**Figure 2:**
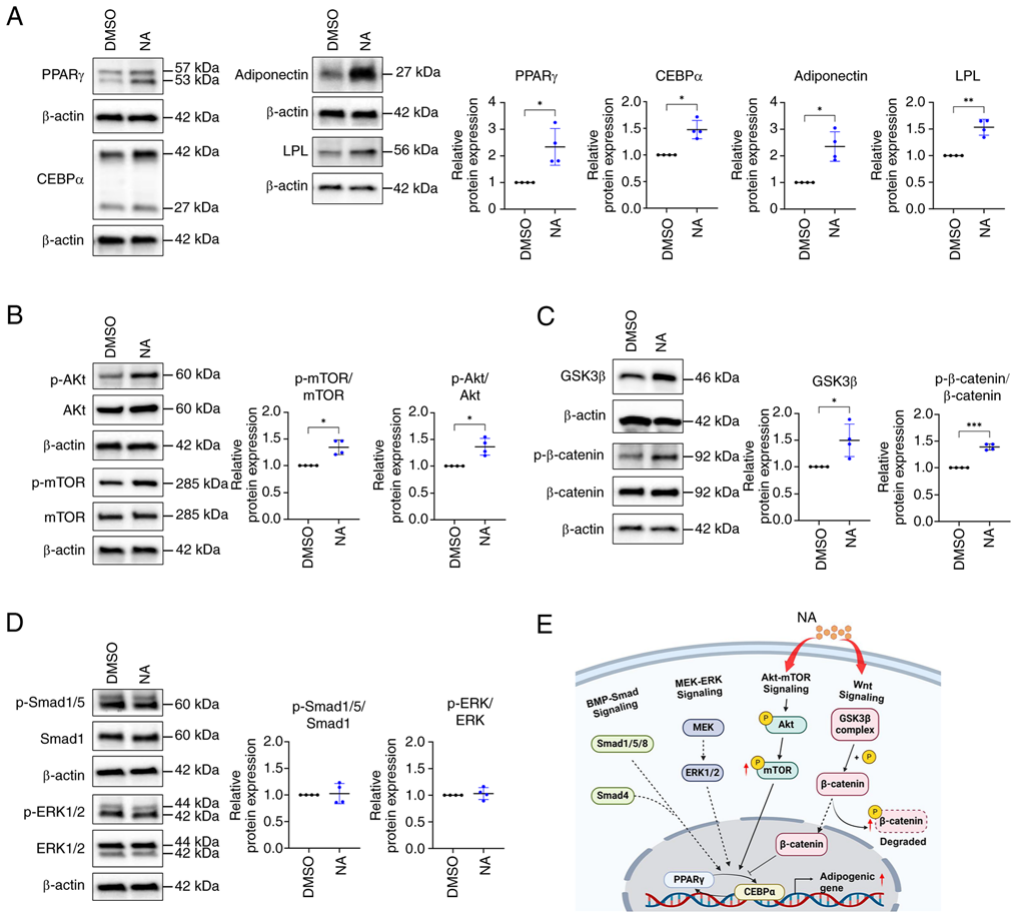
Signaling pathways regulating adipogenesis are affected by NA. (A) Western blot analysis of adipogenic markers after 14 days of adipogenic differentiation and treatment with DMSO or 160 *μ*M NA. (B) Western blot analysis of Akt and mTOR phosphorylation. (C) Western blot analysis of GSK3β expression and β-catenin phosphorylation. (D) Western blot analysis of Smad1/5 and ERK1/2 phosphorylation. (E) Graphical representation of signaling pathways affected by NA during adipogenesis. Created with BioRender.com. Statistical significance was assessed using unpaired Student's t-test: ^*^P<0.05, ^**^P<0.01 and ^***^P<0.001. Data are presented as the mean ± SD. DMSO, dimethyl sulfoxide; LPL, lipoprotein lipase; NA, nervonic acid; p-, phosphorylated.

Because the expression of adipogenic differentiation-related genes was elevated in the NA-treated group, the present study further investigated the expression levels of adipogenesis-related proteins. Consistent with gene expression results, western blotting results indicated that PPARγ, CEBPα, adiponectin and LPL levels were higher in the NA-treated group (2.34±0.69, 1.48±0.17, 2.35±0.55 and 1.53±0.15 fold change, respectively) than in the control group (Fig. 2A). Differentiation of AD-MSCs into mature adipocytes is modulated by the balance of various signaling pathways, such as Smad, MEK/ERK and Akt/mTOR signaling as promoters, and Wnt signaling as an inhibitor (31,32). Since NA promoted the adipogenic differentiation of MSCs, the study aimed to determine the signaling pathway that regulates adipogenesis caused by NA. Western blotting results showed that the phosphorylation of Akt and mTOR, which are positive regulators of adipogenesis, was upregulated after NA treatment (Fig. 2B). Meanwhile, intracellular phosphorylation of β-catenin was significantly upregulated in the NA treatment group (Fig. 2C), indicating that intracellular β-catenin was degraded. Additionally, intracellular GSK3β, which is contained in the destruction complex of β-catenin, was increased (Fig. 2C), which may inhibit Wnt signaling. However, the other positive regulators of adipogenesis, Smad and ERK, were unaffected by NA treatment (Fig. 2D). Collectively, these results indicated that NA may accelerate adipogenesis through activation of the Akt/mTOR pathway and inhibition of Wnt signaling during adipogenesis (Fig. 2E).

### Transcriptome changes regulated by NA during adipogenesis

**Figure 3:**
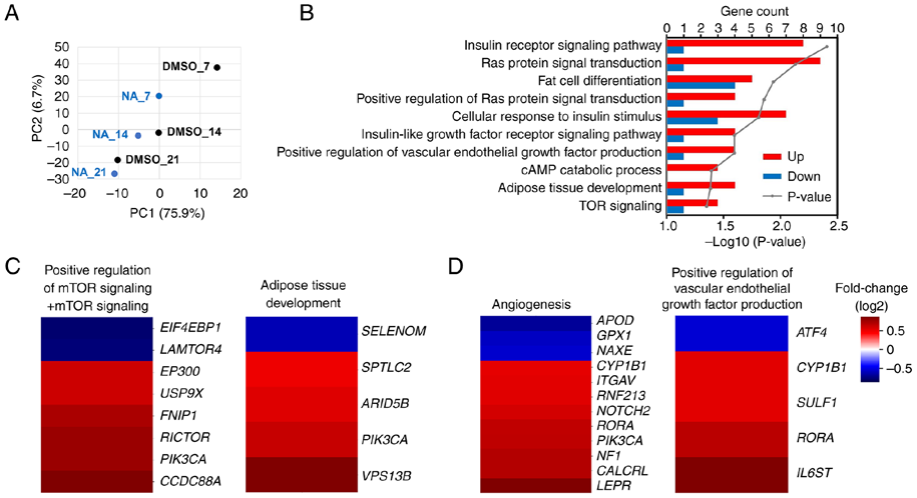
Transcriptomic changes in differentiating AD-MSCs after NA treatment. (A) Principal component analysis of AD-MSCs treated with DMSO or NA during adipogenesis. (B) Gene Ontology analysis of the biological processes associated with differentially expressed genes in the NA-treated group against the DMSO-treated group at day 7 of adipogenesis. (C) Heatmap of the expression of genes involved in 'mTOR signaling, 'positive regulation of mTOR signaling' (left) and 'adipose tissue development' (right) in the NA-treated group versus the DMSO-treated group. (D) Heatmap of the expression of genes involved in 'angiogenesis' (left) and 'positive regulation of vascular endothelial growth factor production' (right) in the NA-treated group versus the DMSO-treated group. AD-MSCs, adipose-derived mesenchymal stem cells; DMSO, dimethyl sulfoxide; NA, nervonic acid.

mRNA sequencing was performed to confirm the transcriptomic changes induced by NA during adipogenesis. PCA implied that genetic changes induced by NA occurred most significantly on day 7 post-adipogenesis induction, and the difference decreased with increasing differentiation period (Fig. 3A). On day 7, 688 genes were upregulated and 387 genes were downregulated in the NA treatment group (fold change >1.5). Gene Ontology analysis revealed that genes upregulated by NA were mainly involved in signaling pathways such as 'GO:0045444~Fat cell differentiation' and 'GO:0060612~Adipose tissue development', which are related to adipogenesis, and 'GO:0008286~Insulin receptor signaling pathway' and 'GO:0031929~TOR signaling', which were partially involved in fat differentiation (Fig. 3B). In addition, the 'hsa04151:PI3K-Akt signaling pathway' and 'hsa04150:mTOR signaling pathway' were included in the signaling cascades affected by NA, which was consistent with the results of protein expression (Figs. S2 and 2B). Furthermore, NA-treated MSCs exhibited upregulated expression patterns for adipose tissue development, mTOR signaling, angiogenesis and VEGF production (Fig. 3C and D). To summarize, NA treatment may enhance Akt/mTOR signaling in MSCs and thereby increase the expression of genes involved in angiogenesis and fat differentiation.

### NA improves adipose tissue engraftment

**Figure 4:**
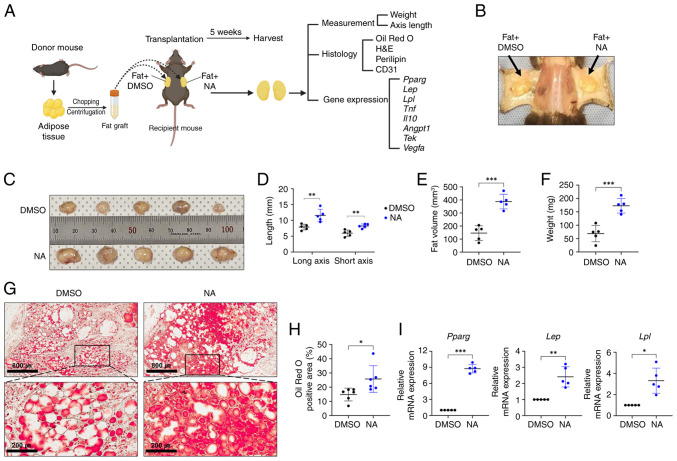
Effects of NA on adipose tissue engraftment. (A) Images of allogeneic fat transplantation in C57BL/6 mice. Peritoneal fat was processed for adipocyte isolation, implanted subcutaneously and treated with 160 *μ*M NA or an equal volume of DMSO. The grafts were harvested after 5 weeks and were further analyzed. Created with BioRender.com. (B) Images of fat grafts 5 weeks after fat transplantation and treatment with DMSO (left) or NA (right). (C) Images and relative sizes of the isolated fat grafts treated with DMSO or NA after transplantation. (D) Axes lengths, (E) fat volume and (F) fat weight of isolated fat grafts treated with DMSO or NA. (G) Oil Red O staining of fat grafts treated with DMSO or NA. Black boxes in the upper images represent the lower images. Scale bars: 800 *μ*m (upper), 200 *μ*m (lower). (H) Oil Red O-positive area within whole fat grafts after transplantation. (I) Reverse transcription-quantitative PCR analysis of *Pparg, Lep* and *Lpl* in the fat grafts. Data are presented as the mean ± SD. Statistical significance was assessed using unpaired Student's t-test: ^*^P<0.05, ^**^P<0.01 and ^***^P<0.001. DMSO, dimethyl sulfoxide; H&E, hematoxylin and eosin; *Lpl*, lipoprotein lipase; NA, nervonic acid.

As aforementioned, it is crucial to create a stable microenvironment within the graft for successful transplantation outcomes (4,5). Since NA demonstrated adipogenesis-promoting effects *in vitro*, the present study further assessed whether NA treatment could improve outcomes after allogeneic fat engraftment in mice. Allogeneic C57BL/6 mouse adipose tissues treated with DMSO or NA were engrafted subcutaneously in mice and histologically analyzed after 5 weeks (Fig. 4A and B). Fig. 4C shows the fat grafts after 5 weeks of fat transplantation (n=5/group). The fat grafts treated with NA showed a significant increase in long and short axes lengths, volume and weight compared with those treated with DMSO (Fig. 4D-F). Lipid accumulation in the fat graft was confirmed by Oil Red O staining. Notably, while the fat grafts treated with DMSO had a number of unstained empty spaces after 5 weeks, the fat grafts treated with NA exhibited much higher staining with completely lipid-filled spaces, indicating that supplementation with NA helped mature adipocytes maintain greater stability after engraftment (Fig. 4G and H). In addition, the mRNA expression levels of *Pparg*, *Lep* and *Lpl* were elevated in the NA-treated fat grafts (Fig. 4I). Taken together, these results suggested that NA may effectively reduce the resorption of transplanted fat and maintain stability after tissue engraftment.

### NA serves a beneficial role in fat engraftment by improving inflammation and angiogenesis

**Figure 5:**
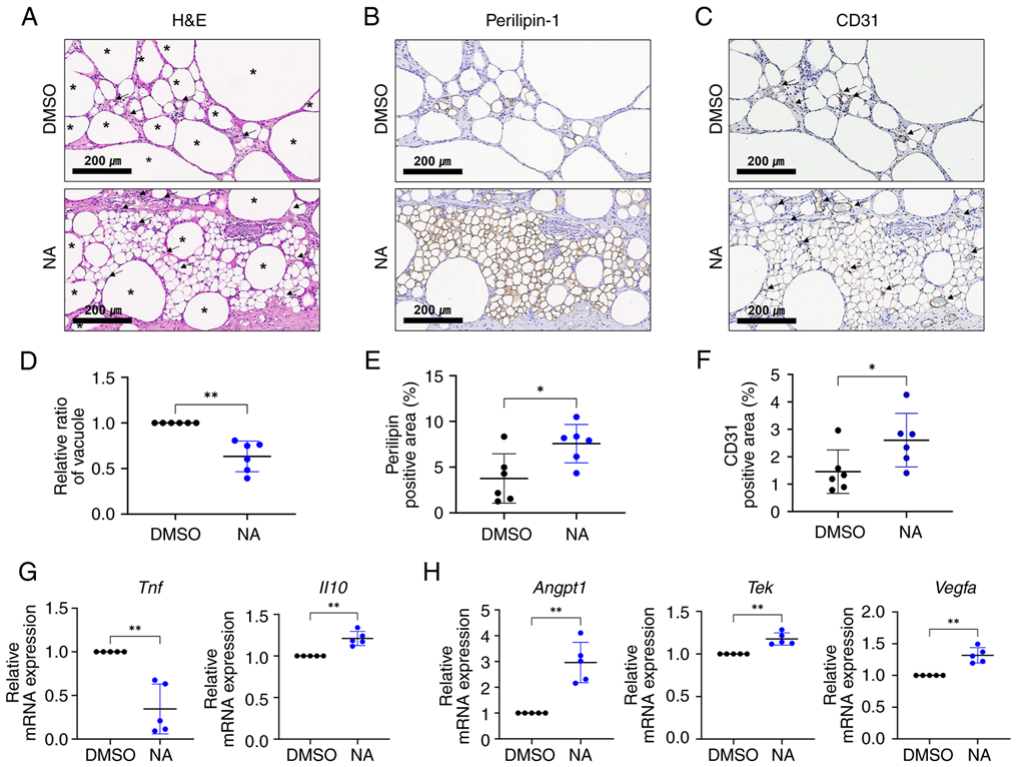
NA functions in fat engraftment by improving inflammation and angiogenesis. (A) H&E staining within grafts treated with DMSO or NA. The black asterisks indicate formed vacuoles or oil cysts within the grafts and the black arrows indicate the vessels within the grafts. Scale bars: 200 *μ*m. Immunohistochemistry analysis of (B) perilipin-1 and (C) CD31 within grafts treated with DMSO or NA. Black arrows indicate the vessel structures within the grafts. Scale bars: 200 *μ*m. The proportion of (D) vacuole area, (E) perilipin-1^+^ area and (F) CD31^+^ area measured within the whole grafts. (G) RT-qPCR analysis of *Tnf* and *Il10.* (H) RT-qPCR analysis of *Angpt1, Tek* and *Vegfa*. Data are presented as the mean ± SD. Statistical significance was assessed using unpaired Student's t-test: ^*^P<0.05 and ^**^P<0.01. DMSO, dimethyl sulfoxide; H&E, hematoxylin and eosin; NA, nervonic acid; RT-qPCR, reverse transcription-quantitative PCR.

Vacuoles (cysts) in fat grafts are formed by the necrosis of adipocytes after fat transplantation, and the proportion of vacuoles in the graft is considered an important indicator for determining the outcome of fat transplantation (33). Therefore, the present study examined vacuole formation in the transplanted fat treated with or without NA. Histological analysis showed that oil cysts or vacuoles with diameters >120 *μ*m (34) were largely formed in the grafts treated with DMSO (Fig. 5A). To confirm the effect of NA on the survival of adipocytes in fat grafts, viable adipocytes were identified using immunohistochemistry for perilipin-1 (Fig. 5B). The fat grafts treated with NA had more perilipin-1^+^ adipocytes with a lower proportion of vacuoles (Fig. 5D and E), verifying that NA may preserve more viable adipocytes. Notably, relatively large portions of small, perilipin-1^+^ adipocytes, which are distinguishable from mature adipocytes, can be seen in the NA-treated grafts (Fig. S3). Subsequently, the degree of graft vascularization was assessed. CD31^+^ vessel structures were more frequently observed in the NA-treated group, with a notable increase in the proportion of the CD31^+^ area (Fig. 5C and F). Moreover, blood vessels in the NA-treated group were relatively evenly distributed between viable adipocytes, improving their survival.

RT-qPCR analysis showed that pro-inflammatory *Tnf* was downregulated, whereas the anti-inflammatory marker *Il10* was elevated (Fig. 5G), and angiogenic-promoting genes *Angpt1*, *Tek* and *Vegfa* were upregulated in grafts supplemented with NA (Fig. 5H). Given that inflammation and neovascularization are critical elements for the successful engraftment of external tissue, these findings indicated that NA may improve the outcomes of adipose tissue engraftment by enhancing angiogenesis and suppressing inflammation, resulting in a relatively well-reorganized microenvironment in the grafts.

## Discussion

In recent years, adipose tissue grafting has been widely used in cosmetic surgery and reconstructive surgery, and various methods or assisting materials for improving the effectiveness and outcome after grafting have emerged, including cell-assisted lipotransfer (6). To the best of our knowledge, the present study was the first to demonstrate that NA could accelerate adipogenic differentiation of human MSCs without cellular toxicity *in vitro*, and could stabilize the graft microenvironment and improve the viability of grafted adipocytes after adipose tissue engraftment *in vivo*. These findings indicated that NA may be a potential candidate for adjuvant therapy in autologous or allogeneic fat transplantation.

In MSCs, cell fate decisions are regulated by a complex interrelated balance of adipogenic and osteogenic transcription factors, cytokines and pathways (35,36). Among them, PI3K/Akt/mTOR signaling activated by insulin or insulin growth factor-1 (IGF1) serves an important role in adipogenesis from an early stage by regulating cell cycle progression and mediating the expression of core transcription factors (37-39). In the present study, the addition of NA during adipogenesis activated the Akt/mTOR pathway. Compared with the control group, AD-MSCs treated with NA exhibited accelerated adipocyte differentiation and upregulated phosphorylation of Akt and mTOR, as determined by western blot analysis. In addition, the expression of signaling molecules involved in the PI3K/Akt pathway and insulin/IGF1 signaling pathway were increased in AD-MSCs treated with NA, as determined by transcriptome analysis. These results indicated that NA may serve as another upstream modulator of insulin/IGF1 pathway molecules during adipogenic induction. Furthermore, NA treatment upregulated the phosphorylation of β-catenin, suppressing the canonical Wnt pathway, which is a core osteogenesis-promoting and anti-adipogenic signaling pathway (40). Thus, NA may tip the cell fate balance toward adipogenesis by activating adipogenic-stimulating signaling and concurrently inhibiting anti-adipogenic molecules.

Additionally, NA may activate other adipogenesis-stimulating factors through the modulation of lipid metabolism. Keppley *et al* (12) reported that dietary NA elevated energy metabolism markers, including PPARα, SIRT1 and PGC1α. Among them, PPARα has been shown to stimulate adipogenic gene expression in both 3T3-L1 preadipocytes and mice when activated by its agonists (41). Moreover, treatment of senescent MSCs with NA may activate the genes *ANGPTL4* and *PLIN2*, which are involved in lipid metabolism (13). Considering these relationships, NA could accelerate adipogenesis not only through direct activation of transcription factors but also through indirect regulation of lipid metabolism.

In allogeneic fat transplantation, NA supplementation with adipose tissue grafts increased lipid accumulation within the grafts and improved the overall viability of adipocytes with fewer vacuoles or cysts. Notably, within the NA-supplemented grafts, there were relatively large portions of small perilipin-1^+^ adipocytes, distinguished from other adipocytes. These small perilipin-1^+^ adipocytes are regenerative adipocytes, which are newly differentiated cells from adipose-derived stem cells (ADSCs) within the graft (42). In fat engraftment using liposuction, while existing adipocytes are largely dead, ADSCs can compensate by proliferating and newly differentiating into adipocytes 2-3 weeks after transplantation (42,43). Considering that the balance between proliferation and regeneration of these newly generated adipocytes against necrosis determines the long-term maintenance of graft volume (44), NA may help improve the viability of existing adipocytes and may stimulate the stable differentiation of ADSCs in the graft.

Furthermore, NA supplementation slightly downregulated the mRNA expression levels of pro-inflammatory cytokines, such as TNF-α, while increasing those of anti-inflammatory factors like IL-10, alleviating inflammation response. Several studies have suggested the anti-inflammatory functions of NA in various diseases, such as colitis and Parkinson's disease, especially in reducing the release of pro-inflammatory cytokines, such as TNF-α, IL-6 and NF-κB with therapeutic effects (45,46). Consistent with these reports, the level of NA has been found to be positively correlated with the concentration of anti-inflammatory adiponectin in epicardial adipose tissue (47). Moreover, adiponectin and IL-10 are adipokines secreted by differentiated adipocytes, and adiponectin can stimulate macrophage production (48,49), indicating the potential anti-inflammatory roles of NA in fat engraftment. In fat transplantation, one of the typical complications is vacuole or oil cyst formation (33). The main causes of cyst formation are fat necrosis and subsequent inflammation, which compromise the overall viability of fat grafts (33,50). In the present study, NA treatment resulted in improved cell viability with lower levels of inflammation and vacuoles, indicating its potential to establish a relatively stable microenvironment for cell survival and regeneration after engraftment.

Supplying oxygen and nutrients through neovascularization in the graft is another key factor that can improve the survival of grafted fat (51). Neovascularization is mainly achieved through the paracrine effects of ADSCs, providing important evidence for MSC-assisted lipotransfer (52). The present results showed that NA treatment activated several genes involved in angiogenesis and VEGF production in human MSCs during adipogenesis. In addition, the CD31^+^ area, representing blood vessels, was largely increased in the NA graft. Moreover, our previous study found that NA treatment of WJ-MSCs stimulated several pro-angiogenic factors (13). Considering these results, NA could function as a stimulator of angiogenesis for ADSCs during fat transplantation. Future studies on fat transplantation using NA-supplemented MSCs could validate this hypothesis.

To the best of our knowledge, the present study is the first to reveal the function of NA as an adjuvant for adipogenesis and fat engraftment. However, the study was limited by the fact that the effect of NA in fat grafting was confirmed after 5 weeks. To address this limitation, further verification of the effectiveness of NA over a longer duration is necessary. Additionally, DMSO, used as a solvent for NA in the present study, is not suitable for injection into humans due to its potential adverse effects; thus, future research should focus on alternative formulations and methods for using NA in clinical practice.

**Figure 6:**
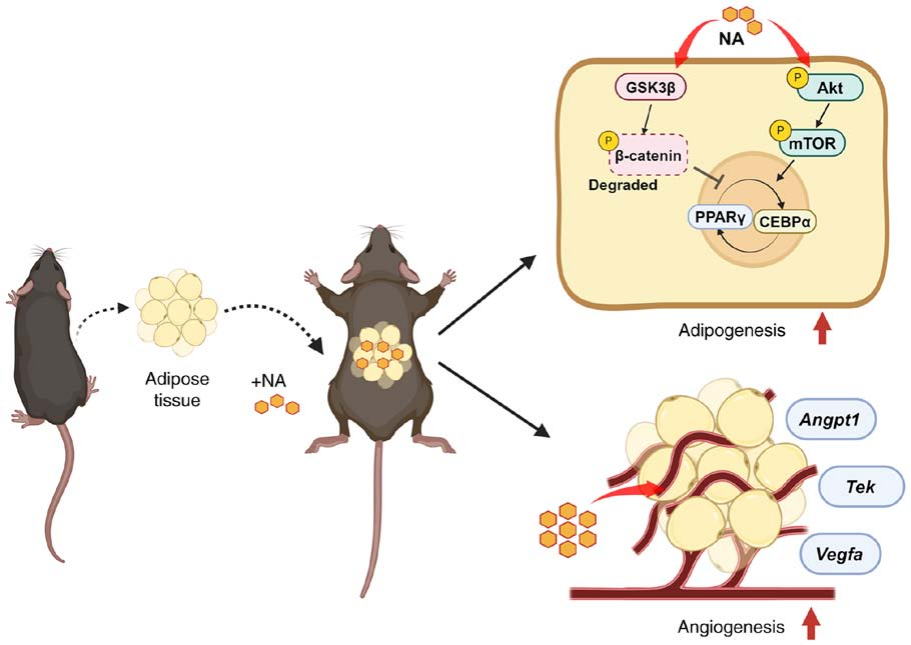
NA is a potential adjuvant strategy for supporting fat grafts in regenerative surgery. The addition of NA to adipose tissue transplantation could accelerate the differentiation of adipocytes through the activation of Akt/mTOR signaling and inhibition of Wnt signaling, and could facilitate the reconstruction of the microenvironment within the transplanted adipose tissue by promoting angiogenesis. Created with BioRender.com. NA, nervonic acid.

In conclusion, the present study demonstrated that NA stimulated and accelerated the differentiation of adipocytes through the activation of Akt/mTOR signaling and inhibition of Wnt signaling. The addition of NA to adipose tissue engraftment helped in the reconstruction of the microenvironment within the fat graft by attenuating inflammation and promoting neovascularization, thus improving the outcome of fat transplantation (Fig. 6). These findings may contribute to the identification of new adjuvants for fat engraftment with better outcomes and higher long-term stability. Additionally, future research aimed at human applications could achieve superior outcomes in regenerative surgery.

## Supplementary Data



## Figures and Tables

**Table I:** Primer sequences used for reverse transcription-quantitative PCR.

Gene	Forward	Reverse
Human *PPARG*	5′-ATGGGTGAAACTCTGGGAGA-3′	5′-TGGAATGTCTTCGTAATGTGGA-3′
Human *CEBPA*	5′-ACTGGGACCCTCAGCCTTG-3′	5′-TGGACTGATCGTGCTTCGTG-3′
Human *ADIPOQ*	5′-AGATCCAGGTCTTATTGGTCC-3′	5′-CTTTCCTGCCTTGGATTCC-3′
Human *LPL*	5′-CATTCCCGGAGTAGCAGAG-3′	5′-ATTCCTGTTACCGTCCAGC-3′
Human *GAPDH*	5′-GAAGGTGAAGGTCGGAGT-3′	5′-TGGCAACAATATCCACTTTACCA-3′
Mouse *Pparg*	5′-GCATTTCTGCTCCACACTAT-3′	5′-CTTTGATCGCACTTTGGTATTC-3′
Mouse *Lep*	5′-TGTGGCTTTGGTCCTATCT-3′	5′-CGACTGCGTGTGTGAAAT-3′
Mouse *Lpl*	5′-GTGGATAAGCGACTCCTACT-3′	5′-TCTCCCTAGCACAGAAGATG-3′
Mouse *Tnf*	5′-CCACGCTCTTCTGTCTACT-3′	5′-CAGGCTTGTCACTCGAATTT-3′
Mouse *Il10*	5′-TTACTGACTGGCATGAGGAT-3′	5′-AGAAAGTCTTCACCTGGCT-3′
Mouse *Angpt1*	5′-GAAGATGGAAGCCTGGATTT-3′	5′-CTGCCTCTGACTGGTTATTG-3′
Mouse *Tek*	5′-TCCCTCCTCAACCAGAAA-3′	5′-TTTGCCCTGAACCTTATACC-3′
Mouse *Vegfa*	5′-CTCTTCTCGCTCCGTAGTA-3′	5′-CCTCTCCTCTTCCTTCTCTT-3′
Mouse *Gapdh*	5′-TTCAACGGCACAGTCAAG-3′	5′-CCAGTAGACTCCACGACATA-3′

## Data Availability

The data generated in the present study may be requested from the corresponding author. The mRNA sequencing data generated in the present study may be found in the GEO under accession number GSE269065 or at the following URL: https://www.ncbi.nlm.nih.gov/geo/query/acc.cgi?acc=GSE269065

## References

[1] Shim YH, Zhang RH (2017). Literature review to optimize the autologous fat transplantation procedure and recent technologies to improve graft viability and overall outcome: A aystematic and retrospective analytic approach. Aesthetic Plast Surg.

[2] Mahoney CM, Imbarlina C, Yates CC, Marra KG (2018). Current therapeutic strategies for adipose tissue defects/repair using engineered biomaterials and biomolecule formulations. Front Pharmacol.

[3] Gao S, Lu B, Zhou R, Gao W (2023). Research progress of mechanisms of fat necrosis after autologous fat grafting: A review. Medicine (Baltimore).

[4] Wu M, Li Y, Wang Z, Feng J, Wang J, Xiao X, Lu F, Dong Z (2020). Botulinum toxin A improves supramuscular fat graft retention by enhancing angiogenesis and adipogenesis. Dermatol Surg.

[5] Major GS, Simcock JW, Woodfield TBF, Lim KS (2022). Overcoming functional challenges in autologous and engineered fat grafting trends. Trends Biotechnol.

[6] Landau MJ, Birnbaum ZE, Kurtz LG, Aronowitz JA (2018). Review: Proposed methods to improve the survival of adipose tissue in autologous fat grafting. Plast Reconstr Surg Glob Open.

[7] Dang J, Yang J, Yu Z, Chen L, Zhang Z, Wang K, Tang J, Yi C (2022). Bone marrow mesenchymal stem cells enhance angiogenesis and promote fat retention in fat grafting via polarized macrophages. Stem Cell Res Ther.

[8] Anderson AE, Wu I, Parrillo AJ, Wolf MT, Maestas DR, Graham I, Tam AJ, Payne RM, Aston J, Cooney CM (2022). An immunologically active, adipose-derived extracellular matrix biomaterial for soft tissue reconstruction: Concept to clinical trial. NPJ Regen Med.

[9] Tang Q, Chen C, Wang X, Li W, Zhang Y, Wang M, Jing W, Wang H, Guo W, Tian W (2017). Botulinum toxin A improves adipose tissue engraftment by promoting cell proliferation, adipogenesis and angiogenesis. Int J Mol Med.

[10] Li Q, Chen J, Yu X, Gao JM (2019). A mini review of nervonic acid: Source, production, and biological functions. Food Chem.

[11] Kageyama Y, Deguchi Y, Hattori K, Yoshida S, Goto YI, Inoue K, Kato T (2021). Nervonic acid level in cerebrospinal fluid is a candidate biomarker for depressive and manic symptoms: A pilot study. Brain Behav.

[12] Keppley LJW, Walker SJ, Gademsey AN, Smith JP, Keller SR, Kester M, Fox TE (2020). Nervonic acid limits weight gain in a mouse model of diet-induced obesity. FASEB J.

[13] Kim SJ, Kwon S, Chung S, Lee EJ, Park SE, Choi SJ, Oh SY, Ryu GH, Jeon HB, Chang JW (2023). Nervonic acid inhibits replicative senescence of human Wharton's Jelly-derived mesenchymal stem cells. Int J Stem Cells.

[14] Palumbo P, Lombardi F, Siragusa G, Cifone MG, Cinque B, Giuliani M (2018). Methods of isolation, characterization and expansion of human adipose-derived stem cells (ASCs): An overview. Int J Mol Sci.

[15] Choi YS, Park YB, Ha CW, Kim JA, Heo JC, Han WJ, Oh SY, Choi SJ (2017). Different characteristics of mesenchymal stem cells isolated from different layers of full term placenta. PLoS One.

[16] Kim JY, Kim DH, Kim DS, Kim JH, Jeong SY, Jeon HB, Lee EH, Yang YS, Oh W, Chang JW (2010). Galectin-3 secreted by human umbilical cord blood-derived mesenchymal stem cells reduces amyloid-beta42 neurotoxicity in vitro. FEBS Lett.

[17] Park SE, Lee J, Chang EH, Kim JH, Sung JH, Na DL, Chang JW (2016). Activin A secreted by human mesenchymal stem cells induces neuronal development and neurite outgrowth in an in vitro model of Alz'eimer's disease: Neurogenesis induced by MSCs via activin A. Arch Pharm Res.

[18] Livak KJ, Schmittgen TD (2001). Analysis of relative gene expression data using real-time quantitative PCR and the 2^−ΔΔCT^ method. Methods.

[19] Andrews S (2010). FastQC: A quality control tool for high throughput sequence data.

[20] Hannon Lab: FASTX-Toolkit (RRID:SCR_005534).

[21] Bushnell B (2014). BBMap.

[22] Trapnell C, Pachter L, Salzberg SL (2009). TopHat: Discovering splice junctions with RNA-Seq. Bioinformatics.

[23] Roberts A, Trapnell C, Donaghey J, Rinn JL, Pachter L (2011). Improving RNA-seq expression estimates by correcting for fragment bias. Genome Biol.

[24] Huang da W, Sherman BT, Lempicki RA (2009). Systematic and integrative analysis of large gene lists using DAVID bioinformatics resources. Nat Protoc.

[25] Huang da W, Sherman BT, Lempicki RA (2009). Bioinformatics enrichment tools: Paths toward the comprehensive functional analysis of large gene lists. Nucleic Acids Res.

[26] Kanehisa M, Sato Y (2020). Kegg mapper for inferring cellular functions from protein sequences. Protein Sci.

[27] Kanehisa M, Sato Y, Kawashima M (2022). Kegg mapping tools for uncovering hidden features in biological data. Protein Sci.

[28] Tomayko MM, Reynolds CP (1989). Determination of subcutaneous tumor size in athymic (nude) mice. Cancer Chemother Pharmacol.

[29] Council NR (2011). Guide for the care and use of laboratory animals.

[30] Bankhead P, Loughrey MB, Fernández JA, Dombrowski Y, McArt DG, Dunne PD, McQuaid S, Gray RT, Murray LJ, Coleman HG (2017). QuPath: Open source software for digital pathology image analysis. Sci Rep.

[31] Ghaben AL, Scherer PE (2019). Adipogenesis and metabolic health. Nat Rev Mol Cell Biol.

[32] Prusty D, Park BH, Davis KE, Farmer SR (2002). Activation of MEK/ERK signaling promotes adipogenesis by enhancing peroxisome proliferator-activated receptor gamma (PPARgamma) and C/EBPalpha gene expression during the differentiation of 3T3-L1 preadipocytes. J Biol Chem.

[33] Mineda K, Kuno S, Kato H, Kinoshita K, Doi K, Hashimoto I, Nakanishi H, Yoshimura K (2014). Chronic inflammation and progressive calcification as a result of fat necrosis: The worst outcome in fat grafting. Plast Reconstr Surg.

[34] Hu Y, Jiang Y, Wang M, Tian W, Wang H (2018). Concentrated growth factor enhanced fat graft survival: A comparative study. Dermatol Surg.

[35] Muruganandan S, Roman AA, Sinal CJ (2009). Adipocyte differentiation of bone marrow-derived mesenchymal stem cells: Cross talk with the osteoblastogenic program. Cell Mol Life Sci.

[36] Chen Q, Shou P, Zheng C, Jiang M, Cao G, Yang Q, Cao J, Xie N, Velletri T, Zhang X (2016). Fate decision of mesenchymal stem cells: Adipocytes or osteoblasts?. Cell Death Differ.

[37] Chang E, Kim CY (2019). Natural products and obesity: A focus on the regulation of mitotic clonal expansion during adipogenesis. Molecules.

[38] Zhang HH, Huang J, Düvel K, Boback B, Wu S, Squillace RM, Wu CL, Manning BD (2009). Insulin stimulates adipogenesis through the Akt-TSC2-mTORC1 pathway. PLoS One.

[39] Tang QQ, Lane MD (2012). Adipogenesis: From stem cell to adipocyte. Annu Rev Biochem.

[40] de Winter TJJ, Nusse R (2021). Running against the Wnt: How Wnt/β-catenin suppresses adipogenesis. Front Cell Dev Biol.

[41] Goto T, Lee JY, Teraminami A, Kim YI, Hirai S, Uemura T, Inoue H, Takahashi N, Kawada T (2011). Activation of peroxisome proliferator-activated receptor-alpha stimulates both differentiation and fatty acid oxidation in adipocytes. J Lipid Res.

[42] Sunaga A, Sugawara Y, Katsuragi-Tomioka Y, Kobayashi E (2013). The fate of nonvascularized fat grafts: Histological and bioluminescent study. Plast Reconstr Surg Glob Open.

[43] Eto H, Kato H, Suga H, Aoi N, Doi K, Kuno S, Yoshimura K (2012). The fate of adipocytes after nonvascularized fat grafting: Evidence of early death and replacement of adipocytes. Plast Reconstr Surg.

[44] Mashiko T, Yoshimura K (2015). How does fat survive and remodel after grafting?. Clin Plast Surg.

[45] Wang X, Liang T, Mao Y, Li Z, Li X, Zhu X, Cao F, Zhang J (2023). Nervonic acid improves liver inflammation in a mouse model of Parkinson's disease by inhibiting proinflammatory signaling pathways and regulating metabolic pathways. Phytomedicine.

[46] Yuan SN, Wang MX, Han JL, Feng CY, Wang M, Wang M, Sun JY, Li NY, Simal-Gandara J, Liu C (2023). Improved colonic inflammation by nervonic acid via inhibition of NF-kappaB signaling pathway of DSS-induced colitis mice. Phytomedicine.

[47] Sawaguchi T, Nakajima T, Hasegawa T, Shibasaki I, Kaneda H, Obi S, Kuwata T, Sakuma M, Toyoda S, Ohni M (2018). Serum adiponectin and TNFalpha concentrations are closely associated with epicardial adipose tissue fatty acid profiles in patients undergoing cardiovascular surgery. Int J Cardiol Heart Vasc.

[48] Wolf AM, Wolf D, Rumpold H, Enrich B, Tilg H (2004). Adiponectin induces the anti-inflammatory cytokines IL-10 and IL-1RA in human leukocytes. Biochem Biophys Res Commun.

[49] Lira FS, Rosa JC, Pimentel GD, Seelaender M, Damaso AR, Oyama LM, do Nascimento CO (2012). Both adiponectin and interleukin-10 inhibit LPS-induced activation of the NF-kappaB pathway in 3T3-L1 adipocytes. Cytokine.

[50] Yoshimura K, Coleman SR (2015). Complications of fat grafting: How they occur and how to find, avoid, and treat them. Clin Plast Surg.

[51] Evans BGA, Gronet EM, Saint-Cyr MH (2020). How fat grafting works. Plast Reconstr Surg Glob Open.

[52] Moustaki M, Papadopoulos O, Verikokos C, Karypidis D, Masud D, Kostakis A, Papastefanaki F, Roubelakis MG, Perrea D (2017). Application of adipose-derived stromal cells in fat grafting: Basic science and literature review. Exp Ther Med.

