# Supplementary material for: Nervonic acid improves fat transplantation by promoting adipogenesis and angiogenesis
Source: Int J Mol Med. 2024 Sep 30;54(6):108. doi: 10.3892/ijmm.2024.5432 (PMC11517738; doi:10.3892/ijmm.2024.5432)

Figure S1. Accelerating effect of NA during adipogenesis on human tissue-derived MSCs. Representative images of Oil-Red O staining and relative amount of lipid accumulation after 7,14 and 21 days of adipogenic differentiation and treatment with DMSO or 160 μM NA in various tissue-derived MSCs: (A) UCB-MSCs, (B) PL-MSCs, (C) WJ-MSCs and (D) BM-MSCs. Scale bars: 100 μm. Data are presented as the mean ± SD. Statistical significance was assessed using unpaired Student's t-test: $^{*}P<0.05$, $^{**}P<0.01$, $^{***}P<0.001$. BM, bone marrow; DMSO, dimethyl sulfoxide; MSCs, mesenchymal stem cells; NA, nervonic acid; PL, placenta; UCB, umbilical cord blood; WJ, Wharton's jelly.

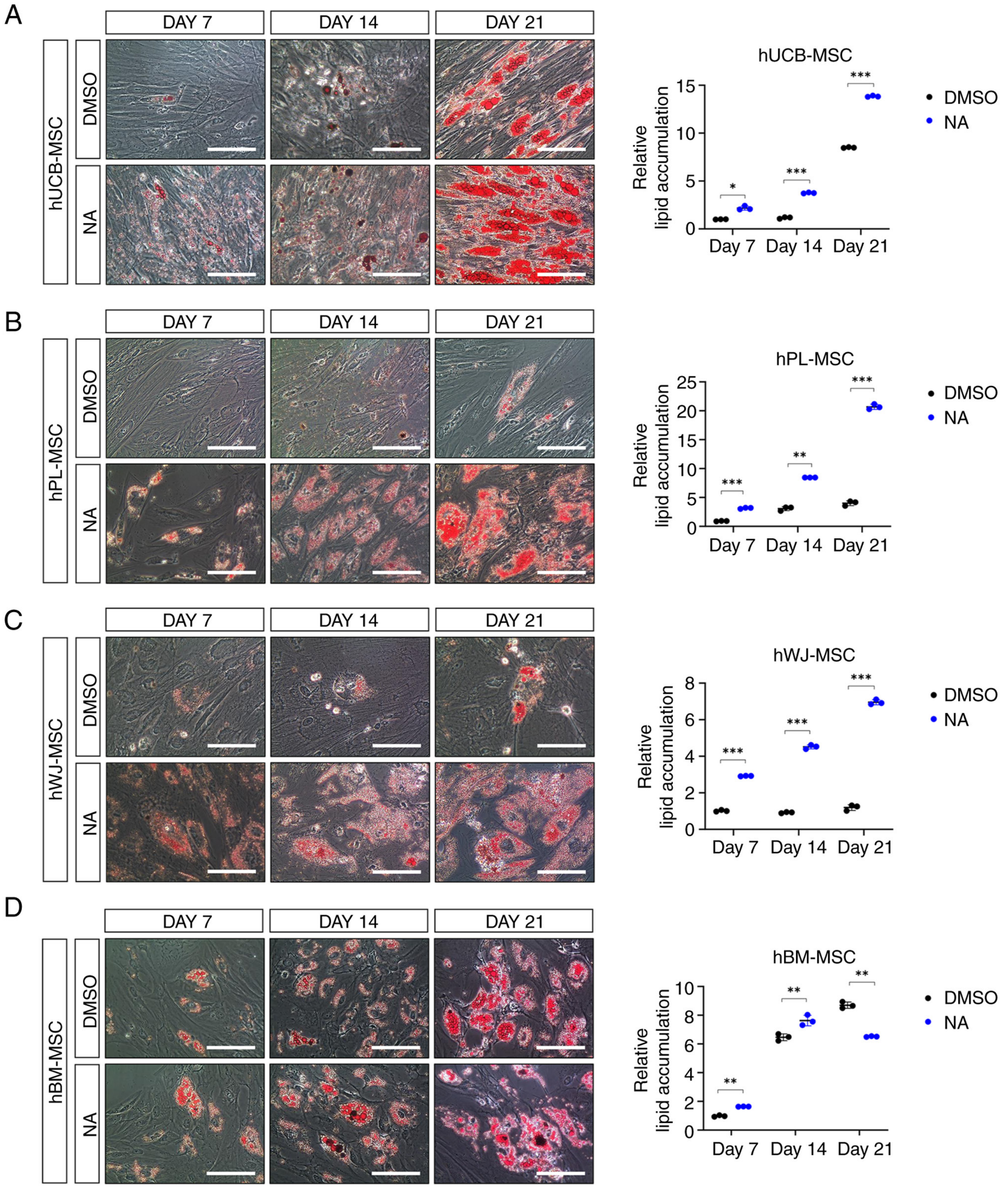

Figure S2. Signaling pathways and their components affected by NA. Changes in expression of molecules involved in the (A) PI3K/Akt and (B) mTOR pathways after 7 days of adipogenic differentiation and treatment with 160 μM NA versus dimethyl sulfoxide. Signaling pathways related to the selected DEGs were analyzed using the KEGG mapper. Red boxes indicate upregulated moleculesand blue boxes indicate downregulated molecules. NA, nervonic acid.

A

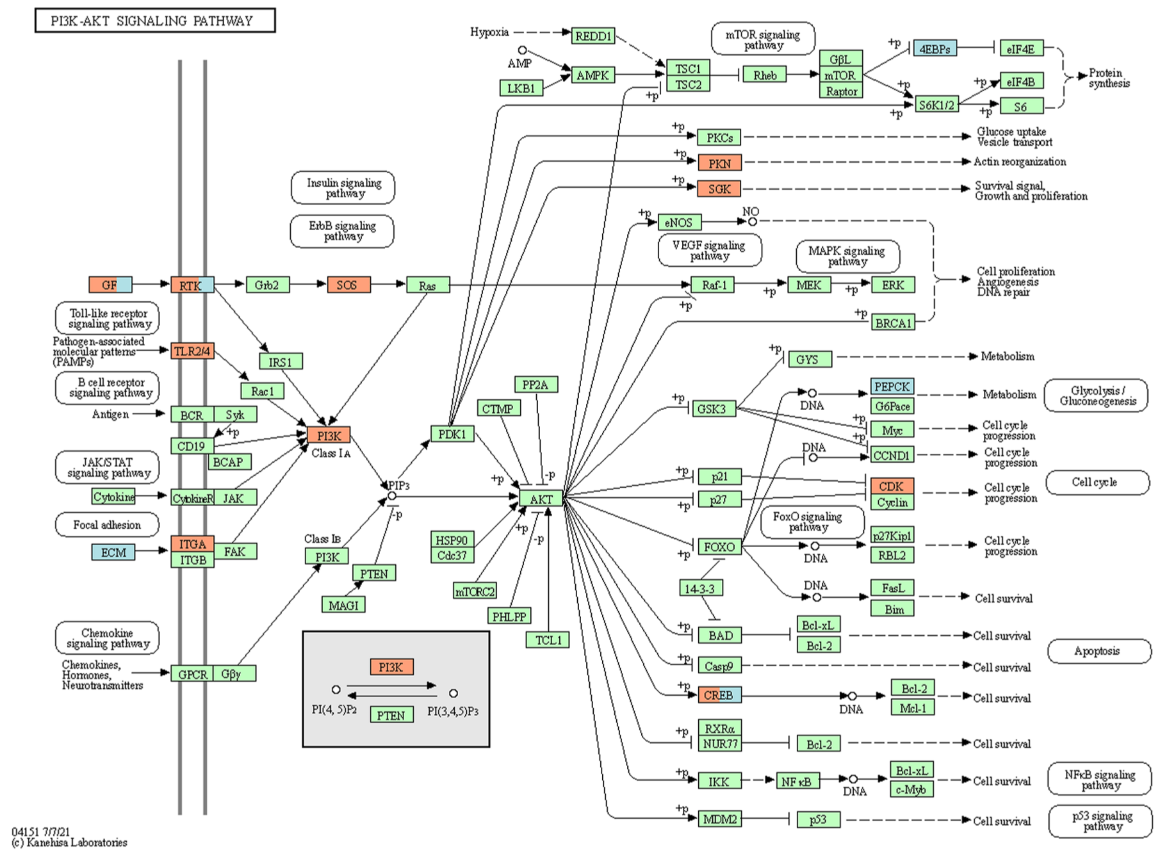

B

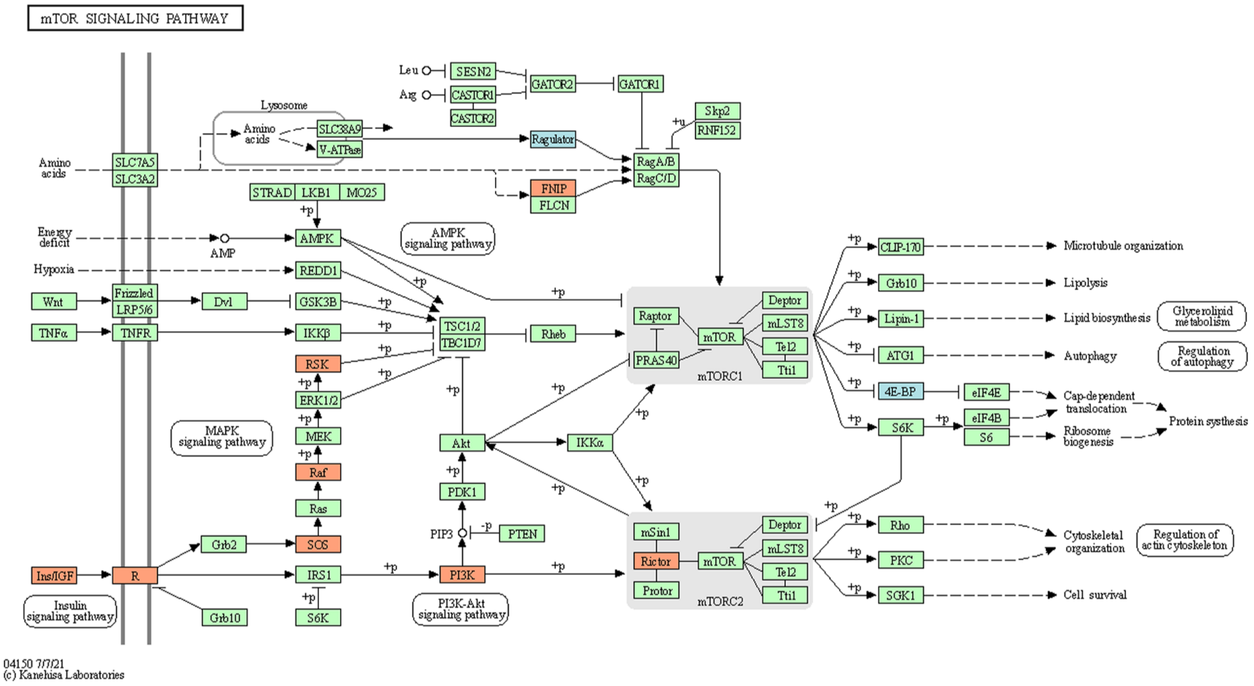

Figure S3. Small perilipin-1^{+} adipocytes in the fat graft treated with NA. Immunohistochemical images of perilipin-1 in grafts treated with NA. Black arrows indicate small perilipin-1^{+} adipocytes, which represent regenerative adipocytes derived from adipose-derived stem cells in the grafts. Scale bars: 100 μm. NA, nervonic acid.

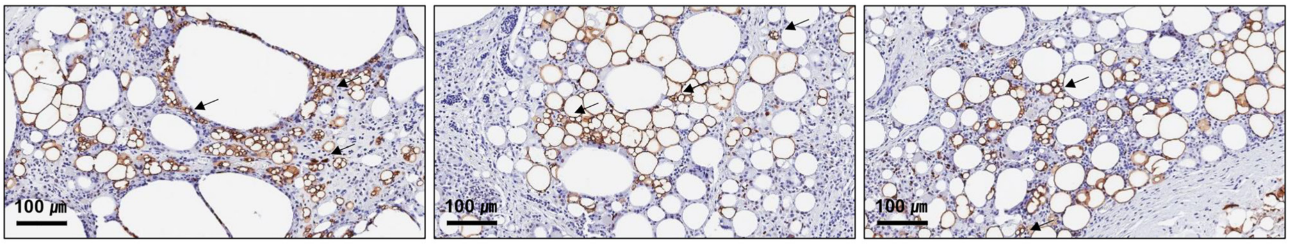

Supplement: Supplementary file 1 [file Supplementary_Data.pdf]
